# Applying Administrative Linkage to Longitudinal Aging Studies: Boston Early Adversity and Mortality Study

**DOI:** 10.1093/geroni/igaa057.1269

**Published:** 2020-12-16

**Authors:** Mina Antic, Ashley Dorame, Joseph Ferrie, Maria Lopes, Robert Waldinger, Avron Spiro, Daniel Mroczek, Lewina Lee

**Affiliations:** 1 Massachusetts General Hospital, Boston, Massachusetts, United States; 2 Boston University School of Medicine, Boston, Massachusetts, United States; 3 Northwestern University, Evanston, Illinois, United States; 4 VA Boston Healthcare System, Waban, Massachusetts, United States; 5 Northwestern University, Chicago, Illinois, United States

## Abstract

Adverse childhood experiences have been linked to poor adult health, yet the underlying pathways remain unclear. While longitudinal aging studies provide rich data on health trajectories in adulthood, two intrinsic limitations hamper progress in studying causal pathways: (1) reliance on retrospective assessment of early-life conditions, and (2) inadequate data coverage on lifespan developmental processes, especially in childhood. The Boston Early Adversity and Mortality Study (BEAMS) was designed to overcome these limitations by applying high-quality administrative record linkage to three longitudinal studies on aging that are over 50-years-old. BEAMS uses administrative linkage to acquire contemporaneous, early-life information on health, family, and environmental hazards from multiple databases. Our sample includes male participants from the VA Normative Aging (n=2280), Grant (n=456), and Glueck (n=268) Studies. BEAMS extends linkage to siblings, thus including women, so that our combined sample is representative of the early 1900s Northeastern U.S. population. Key steps in administrative linkage include coding identifiers from existing data; linkage to 1900-40 Censes, vital, and military (WWI, WWII, Veterans benefits) records; linkage to public databases for early-life lead exposure data, and later-life health information (Medicare, NDI). By linking records of study participants (74%-94% deceased) to numerous administrative databases, BEAMS will create a cradle-to-grave dataset with prospective data on early socioeconomic, psychosocial, and environmental exposures, and lifespan health data. BEAMS uses human review to achieve high-quality record linkage. Our methodology can be adopted by other longitudinal aging studies to overcome barriers in advancing causal knowledge on pathways linking early-life conditions to lifespan health outcomes.

